# Molecular Epidemiology of Dengue Virus Strains from Finnish Travelers

**DOI:** 10.3201/eid1401.070865

**Published:** 2008-01

**Authors:** Eili Huhtamo, Nathalie Y. Uzcátegui, Heli Siikamäki, Auli Saarinen, Heli Piiparinen, Antti Vaheri, Olli Vapalahti

**Affiliations:** * Haartman Institute, University of Helsinki, Finland; † Helsinki University Central Hospital, Finland; ‡ HUSLAB Hospital District of Helsinki and Uusimaa, Finland; § Faculty of Veterinary Medicine, University of Helsinki, Finland

**Keywords:** Dengue virus, isolation, traveler, dispatch

## Abstract

We characterized 11 dengue virus (DENV) isolates obtained from Finnish travelers during 2000–2005 using monoclonal antibodies and phylogenetic analysis. The analysis of DENV isolated from travelers contributes to the global picture of strain distribution and circulation. The isolates included all serotypes, including a DENV-2 isolate from Ghana.

## Abstract 2

Molecular Epidemiology of Dengue Virus Strains from Finnish Travelers

Dengue viruses (DENV 1–4) are mosquito-borne members of the family Flaviviridae, genus *Flavivirus*. Dengue is regarded as the most significant arboviral disease in the world. Disease incidence and prevalence are rising in dengue-endemic areas, and travelers are increasingly affected. The disease can vary from asymptomatic to febrile disease, classic dengue fever, or complications such as dengue hemorrhagic fever or dengue shock syndrome. Several virus- and host-specific factors have been suggested to correlate with severe disease outcomes, which are mostly associated with secondary infections (*1*). These outcomes are not common in European travelers, and deaths are rare (*2*). In recent years, the number of annually diagnosed cases has increased in Finland from an average of 10 to >20 in 2006 (Huhtamo et al., unpub. data). In the present study, study samples collected during 1999–2005 were studied by virus isolation. Virus isolates were not obtained from year 1999 samples; all isolates obtained from these samples were from the years 2000–2005.

## The Study

Patients returning from dengue-endemic areas with fever and other symptoms compatible with dengue were treated mainly at university hospitals in Finland. Because of clinical suspicion, serum samples were tested for antibodies to DENV at Helsinki University Central Hospital Laboratory. The diagnosis was based on detection of immunoglobulin (Ig) M in the acute- or convalescent-phase sample or on a 4-fold IgG titer rise in paired serum specimens in an in-house IgG immunofluorescence assay (IFA), and IgM-enzyme immunoassay (Focus Technologies, Cypress, CA, USA). For this study, serum specimens from all patients were aliquoted and stored at –70ºC.

From patients with dengue diagnosis, acute-phase serum specimens with IgG titers <320 (IFA) were chosen for virus isolation (n = 40). Virus isolations were done simultaneously in 2 cell lines: in Vero E6 cells (ATCC CRL-1586) grown in minimal essential medium at 37°C and 5% CO_2_, and in C6/36 *Aedes albopictus* cells (ATCC CRL-1660) grown in Leibowitch L-15 medium at room temperature. Cells in 25-cm^2^ flasks were incubated with 50 μL of patient serum for 1 hour and observed for 24 days for cytopathic effects (CPEs). When CPEs were evident, cells were harvested for IFA, and RNA was extracted from supernatants for reverse transcriptase–PCR (RT-PCR). In the absence of CPEs, cells were subcultured after 7 days into 75-cm^2^ culture flasks and studied by IFA on days 7 and 24.

In IFA, the cells were stained with a DENV-positive serum and DENV–type-specific monoclonal antibodies (MAbs) (*3*). RNA was extracted from IFA- or CPE-positive culture supernatants with a Viral RNA Mini Kit (QIAGEN, Valencia, CA, USA) according to the manufacturer’s instructions. RT-PCR targeting the capsid–premembrane (C-preM) region was performed using DENV-specific primers (*4*), Expand reverse transcriptase (Roche, Basel, Switzerland) and Taq DNA polymerase (Fermentas, Glen Burnie, MD, USA).

A total of 11 DENV strains were isolated from different geographic locations, including the 4 serotypes (DENV-1, n = 4; DENV-2, n = 2; DENV-3, n = 3; DENV-4, n = 2; Table). The serum samples yielding virus isolates were drawn within 1 week after onset of symptoms, which included fever, headache, muscular pain, rash, and nausea. Most of these samples were positive for antibodies to DENV (IgM positive, n = 8; IgG positive, n = 5).

**Table:** Dengue virus isolates from Finnish travelers, 2000–2005*

Virus serotype	Patient travel history	Year	Isolate/ case no.	Strain designation (GenBank accession no.)	IFA screening of infected cells†						Isolation serum antibody status			Patient sex/ age, y
					Vero E6			C6/36						
					dpi	Pos		dpi	Pos		dpo	IgM	IgG	
DENV-1	Thailand	2002	3	F9.D1.02 (EU005250)	7	+++		7	++		5	–	<10	F/23
DENV-1	Malaysia/ Thailand	2002	4	F12.D1.02 (EU005249)	–	–		24	+		7	+	20	F/43
DENV-1	Thailand	2005	8	F31.D1.05 (EU005248)	–	–		24	+		7	+	<10	F/56
DENV-1	India	2005	11	F37.D1.05 (EU005247)	5	+++		5	+++		3	NA	NA	M/31
DENV-2	Sri Lanka	2003	6	F18.D2.03 (EU005252)	–	–		24	+		5	+	320	M/54
DENV-2	Ghana	2005	9	F32.D2.05 (C-preM, EU005251; E, EU005258)	7	+++		7	+		2	+	<10	F/22
DENV-3	Cuba	2002	2	F7.D3.02 (EU005253)	7	+		24	+		6	+	20	M/55
DENV-3	Brazil	2003	5	F13.D3.03 (EU005254)	–	–		24	+		5	+	<20	M/26
DENV-3	Sri Lanka	2004	7	F24.D3.04 (EU005255)	4	+++		24	+		2	–	20	F/39
DENV-4	Sri Lanka	2000	1	F2.D4.00 (EU005256)	10	+++		10	+		4	+	<10	F/42
DENV-4	Indonesia	2005	10	F34.D4.05 (EU005257)	–	–		24	+		5	+	20	M/37

*IFA, immunofluoresence assay; dpi, day postinfection; Pos, proportion of positive cells; dpo, day post-onset; Ig, immunoglobulin; DENV, dengue virus; NA, not available; c-preM, capsid–premembrane; E, envelope. † +, ++, and +++ indicate the relative amount of positive cells observed in IFA using fluorescence microscope: individual positive cells observed <10% (+), 10–50% of the cells positive (++), 50%–100% of the cells positive (+++). –, not detected.

Isolates were either strains that grew in both of the tested cell lines (n = 6) or strains that grew only in C6/36 cells (n = 5). Two of the DENV-3 isolates (2 and 7) were detectable considerably earlier in Vero E6 than in C6/36 cells. DENV-1 isolates showed 2 distinct growth patterns; isolates 4 and 8 grew only in C6/36 cells, and isolates 3 and 11 grew in both tested cell lines (Table).

All isolates were successfully serotyped with the RT-PCR of Lanciotti et al. (*4*), in agreement with results of the MAb IFA. However, isolate 3 (DENV-1) had particular properties in type-specific MAb IFA, depending on the cell type because it showed positive results in infected C6/36 cells and negative results in infected VE6 cells.

First-round RT-PCR amplicons were purified by using ExoSAP-IT (US Biochemicals, Cleveland, OH, USA), and directly sequenced. When necessary, the envelope gene was amplified using previously described primers (*5*) and sequenced. Nucleotide sequences of the isolates were aligned with published DENV sequences from GenBank (Appendix Table) using ClustalW (www.ebi.ac.uk/tools/clustalw). Phylogenetic analysis was performed by the neighbor-joining method with a Kimura 2-parameter model using MEGA3 software version 3.1 (*6*).

**Figure 1:**
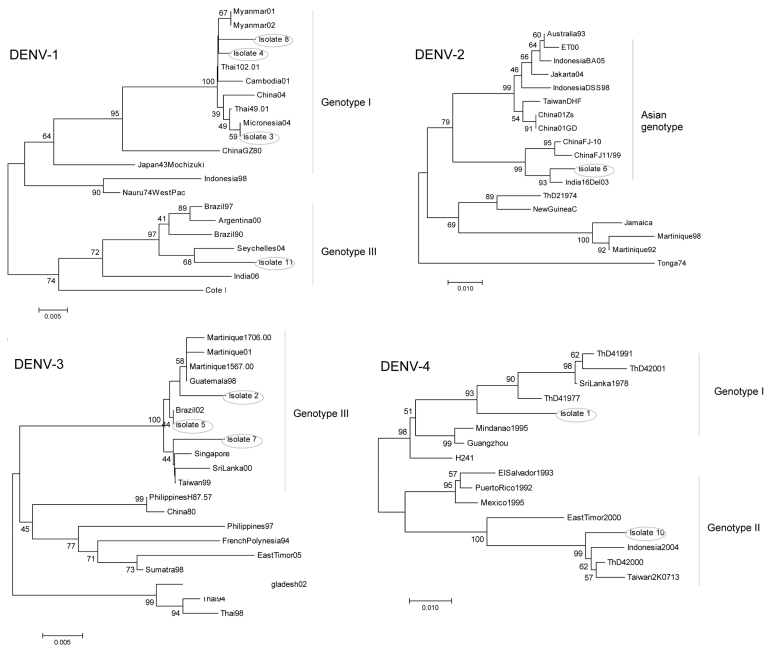
Neighbor-joining phylogenetic trees of the 4 dengue virus (DENV) serotypes based on the 454-bp capsid–premembrane (C-preM) region sequences obtained from first-round amplicons (*6*). Isolates described in this study are circled. Bars represent nucleotide substitutions/site.

**Figure 2:**
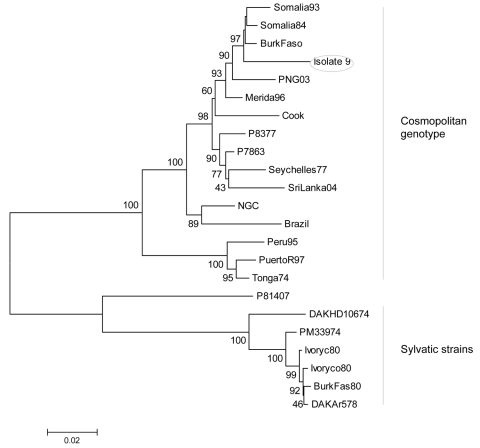
Neighbor-joining phylogenetic tree of dengue virus type 2 (DENV-2) based on the envelope gene sequence (1,485 bp). Isolate 9 from Ghana is circled. Bar represents nucleotide substitutions/site.

Phylogenetic analyses (Figure 1) showed that isolates 3, 4, and 8 (DENV-1) clustered with Asiatic DENV-1 strains of genotype I (*7*), which corresponded with the patients’ travel history. Isolate 11 (DENV-1) from India clustered with a genotype III strain isolated a year earlier from the Seychelles. Isolate 6 (DENV-2), obtained from Sri Lanka in 2003, clustered with a strain isolated in the same year from India. Unlike the other isolates, isolate 9 (DENV-2), obtained in Ghana in 2005, did not group with any of the representative strains of the C-preM region, for which no African sequences were available in GenBank. The additionally studied envelope gene sequence grouped with previous African isolates of the cosmopolitan genotype (*8*) (Figure 2).

The DENV-3 isolates represented genotype III (*9*) (Figure 1). Isolate 2 from Cuba clustered with strains from Martinique in agreement with previous data on Cuban strains (*10*). Isolate 7 (DENV-3), obtained in Sri Lanka in 2004, clustered with strains from Singapore, Sri Lanka, and Taiwan. Isolate 5 was identical in sequence to a strain isolated 1 year earlier from a patient in Brazil who died (*11*). DENV-4 isolates represented 2 different genotypes; isolate 1 from Sri Lanka clustered with genotype I strains, and isolate 10 from Indonesia clustered with genotype II (*12*).

## Conclusions

Studies on imported DENV have provided interesting insights to the global picture of circulating strains (*13**,**14*), and also have led to the discovery of novel DENV strains and lineages (*15**,**16*). In this study, we characterized 11 strains of DENV isolated from Finnish travelers in 2000–2005 and provided new information about strains circulating in India, Sri Lanka, and Ghana.

Previous studies have shown that DENV isolation is possible when antibody levels are low (*17*). However, in this study, most samples yielding virus isolates were antibody positive. The patients had primary infections, except for 1 patient, who had an IgG titer of 320 in the acute phase, which is suggestive of a secondary infection. This was the only patient with any bleeding symptoms, i.e., prolonged bleeding from the venopuncture site.

Virus isolates from Finnish travelers were heterogeneous. All patients had dengue fever, including the patient whose isolate was identical in sequence to a strain isolated from a patient who had died. Since the disease outcomes of the patients were uneventful, no associations could be made between the infective virus serotype or strain and disease severity.

Both mammalian and mosquito cells were used in virus isolation, which enabled the detection of other flaviviruses that may have caused seropositivity through cross-reaction. All DENV isolates grew in C6/36 mosquito cells; however, use of 2 cell lines showed variation in the growth patterns of the isolates in different cell types. We observed that some DENV-3 strains were detectable earlier in mammalian Vero E6 cells than in C6/36 cells, which suggested a different capability to infect these cells. This property could not be associated with pathogenicity in this study; thus, the biologic relevance of this phenomenon is unknown.

The DENV type-specific MAb IFA showed that one of the DENV-1 isolates (isolate 3) had distinct antigenic properties when cultured in mammalian or mosquito cells. Whether this strain represents MAb-escape properties requires further studies.

The phylogenetic grouping of the isolates was consistent with the travel history of the patients in most cases. However, isolate 11 (DENV-1) from India clustered with a genotype III strain isolated a year earlier from the Seychelles, which suggested strain transfer between these countries.

Phylogenetic analysis of isolate 9 (Ghana 2005) showed that it could be grouped with other African isolates of the cosmopolitan genotype (Figure 2). To our knowledge, this is the first DENV-2 strain characterized from Ghana (the geographically nearest isolate is from Burkina Faso in 1983). This grouping demonstrates sustained circulation of DENV-2 strains in Africa for decades.

The 11 DENV isolates represent a random sample from different geographic locations. Three strains were isolated from travelers returning from Sri Lanka, first in 2000 (DENV-4), followed by isolates in 2003 (DENV-2) and 2004 (DENV-3). These strains demonstrate extensive DENV serotype cocirculation.

## Supplementary Material

Appendix Table Dengue virus (DENV) sequences used in the phylogenetic analysis of isolates from Finnish travelers, 2000–2005*
