# Supplementary material for: Molecular Epidemiology of Dengue Virus Strains from Finnish Travelers
Source: Emerg Infect Dis. 2008 Jan;14(1):80–3. doi: 10.3201/eid1401.070865 (PMC2600155; doi:10.3201/eid1401.070865)
Supplement: Appendix Table — Dengue virus (DENV) sequences used in the phylogenetic analysis of isolates from Finnish travelers, 2000–2005* [file 07-0865_appT-s1.pdf]

Appendix Table. Dengue virus (DENV) sequences used in the phylogenetic analysis of isolates from Finnish travelers, 2000–2005*

Abbreviation in phylogenetic tree	Location of origin	Strain	Serotype	Year of isolation	GenBank accession no.
Seychelles04	Seychelles	Reunion 191/04	DENV-1	2004	DQ285559
Brazil97	Brazil	BR/97-409	DENV-1	1997	AF311957
Argentina00	Argentina	295arg00	DENV-1	2000	AF514885
Cambodia01	Cambodia	DENV-1/KHM/2001 L1117016	DENV-1	2001	AF538026
Thai49.01	Thailand	ThD1_0049_01	DENV-1	2001	AY732482
Myanmar01	Myanmar	D1.Myanmar.059/01	DENV-1	2001	AY708047
China04	China	Fj231/04 (Fujian)	DENV-1	2004	DQ193572
Thai102.01	Thailand	ThD1_0102_01	DENV-1	2001	AY732479
Micronesia04	Micronesia	Yap State	DENV-1	2004	AB178040
Myanmar02	Myanmar	D1.Myanmar.49440/02	DENV-1	2002	AY726553
Japan43Mochizuki	Japan	Mochizuki	DENV-1	1943	AB074760
ChinaGZ80	China	GZ80	DENV-1	1980	AF350498
Nauru74WestPac	Nauru	Nauru Island, Western Pacific, clone: WestPac	DENV-1	1974	U88535
Cote D' Ivoire98	Cote d'Ivoire	Abidjan	DENV-1	1998	AF298807
Brazil90	Brazil	Den1BR/90	DENV-1	1990	AF226685
Indonesia98	Sumatra, Indonesia	Strain 98901530	DENV-1	1998	AB189121
India06	India	06/1/del2006	DENV-1	2006	EF127001
BurkFaso	Burkina Faso	Strain 0190	DENV-2	1983	L10042
Somalia93	Somalia	S9/Somalia/93	DENV-2	1993	DQ341119
Somalia84	Somalia	Strain 10	DENV-2	1984	L10051
PNG03	Papua New Guinea	Human	DENV-2	2003	AY706002
Merida96	Mexico	BC17/Merida96	DENV-2	1996	AY449677
Cook	Cook Islands	Cook Islands 1	DENV-2	1997	AF004020
P7863	Malaysia	P7-863	DENV-2	1969	AF231716
Seychelles77	Seychelles	Sey52	DENV-2	1977	L10048
SriLanka04	Sri Lanka	D2/Hu/SriLanka/NIID23/2004	DENV-2	2004	AB194883
P8377	Malaysia	P8-377	DENV-2	1969	AF231715
NGC	Papua New Guinea	New Guinea C	DENV-2	1944	D00346
Brazil	Brazil	BEL63650	DENV-2	NA	AY775307
Peru95	Peru	IQT-1950 Peru 1995	DENV-2	1995	DQ917242
PuertoR97	Puerto Rico	Isolate 1328	DENV-2	1977	DQ917243
Tonga74	Tonga	Tonga 1974	DENV-2	1974	AY744147
DAKHD10674	Senegal	DAKHD10674	DENV-2	1970	AF231720
PM33974	Republic of Guinea	PM33974	DENV-2	1981	AF231719
Ivoryc80	Côte d'Ivoire	1980 DAK Ar A1247	DENV-2	1980	DQ917245
BurkFas80	Burkina Faso	1980 DAK Ar 2039	DENV-2	1980	DQ917246
DAKAr578	Côte d'Ivoire	DAKAr578	DENV-2	1980	AF231718
Ivoryco80	Côte d'Ivoire	DAK Ar510	DENV-2	1980	DQ917244
P81407	Malaysia	P8-1407	DENV-2	1970	AF231717
China01Zs	China	ZS01/01	DENV-2	2001	EF051521
China01GD	China	GD19/2001	DENV-2	2001	AF509530
TaiwanDHF	Taiwan	Taiwan-1008DHF	DENV-2	NA	AY776328
IndonesiaDSS98	Indonesia	98900666 DSS DV-2	DENV-2	1998	AB189124
Jakarta04	Jakarta, Indonesia	TB16i	DENV-2	2004	AY858036
IndonesiaBA05	Jakarta, Indonesia	BA05i	DENV-2	NA	AY858035
Australia93	Australia	TSV01	DENV-2	1993	AY037116
ET00	East Timor	ET300	DENV-2	2000	EF440433
ChinaFJ-10	China	FJ-10	DENV-2	NA	AF276619
ChinaFJ11/99	China	FJ11/99	DENV-2	1999	AF359579
India16Del03	India	16DEL03	DENV-2	2003	AY706095
ThD21974	Thailand	ThD2_0038_74	DENV-2	1974	DQ181806
Jamaica	Jamaica	Jamaica/N.1409	DENV-2	NA	M20558
Martinique98	Martinique	DEN2/H/IMTSSA-MART/98-703	DENV-2	1998	AF208496
Martinique92	Martinique	MAR_92	DENV-2	1992	DQ364519
Thai94	Thailand	C0360/94	DENV-3	1994	AY923865
Thai98	Thailand	KPS-4-0657/207	DENV-3	1998	AY912458
FrenchPolynesia94	French Polynesia (Raiatea)	PF94/136116	DENV-3	1994	AY744685
Taiwan99	Taiwan	99TW268	DENV-3	1999	DQ675533
Philippines97	The Philippines	PhMH-J1-97	DENV-3	1997	AY496879
Bangladesh02	Bangladesh	BDH02-7	DENV-3	2002	AY496877
EastTimor05	East Timor	D3/Hu/TL129NIID/2005	DENV-3	2005	AB214882
Martinique1567.00	Martinique	D3/H/IMTSSA-MART/2000/1567	DENV-3	2000	AY099338
Guatemala98	Guatemala	GUATE98-2	DENV-3	1998	AB038475
Brazil02	Brazil	BR74886/02	DENV-3	2002	AY679147

Martinique01	Martinique	D3/H/IMTSSA-MART/2001/2012	DENV-3	2001	AY099340
Taiwan99	Taiwan	99TW628	DENV-3	1999	DQ675533
Singapore	Singapore	NA	DENV-3	NA	AY662691
SriLanka00	Sri Lanka	D3/H/IMTSSA-SRI/2000/1266	DENV-3	2000	AY099336
PhilippinesH87.57	The Philippines	H87	DENV-3	1957	M93130
Martinique1706.00	Martinique	D3/H/IMTSSA-MART/2000/1706	DENV-3	2000	AH011664
China80	China	80-2	DENV-3	1980	AF317645
Sumatra98	Sumatra, Indonesia	98902890 DF D3	DENV-3	1998	AB189128
H241	The Philippines	H241	DENV-4	1956	AY947539
Guangzhou	Guangzhou, China	B5	DENV-4	NA	AF289029
SriLanka1978	Sri Lanka	No.17/Sri Lanka/1978/Human	DENV-4	1978	AY550909
ThD41991	Thailand	ThD4_0348_91	DENV-4	1991	AY618990
Mindanao1995	The Philippines	Mindanao BDJ	DENV-4	1995	AF177542
ThD42001	Thailand	ThD4_0485_01	DENV-4	2001	AY618992
Mexico1995	Mexico	D4111_1995MX	DENV-4	1995	AY152304
PuertoRico1992	Puerto Rico	D4.34_1992	DENV-4	1992	AY152204
ThD400	Thailand	ThD4_0734_00	DENV-4	2000	AY618993
Taiwan2K0713	Taiwan	Taiwan-2K0713	DENV-4	NA	AY776330
Indonesia2004	Indonesia	SW38i	DENV-4	2004	AY858050
EastTimor2000	East Timor	ET288	DENV-4	2000	EF440435
ElSalvador1993	El Salvador	D4.110_1993ES	DENV-4	1993	AY152300
ThD41977	Thailand	ThD4_0087_77	DENV-4	1977	AY618991

*NA, not available.
